# Use of alternative components to facilitate timely administration of tagraxofusp

**DOI:** 10.1177/10781552251409127

**Published:** 2025-12-24

**Authors:** Richard L Fong, Chase Ayres, Roy Browne

**Affiliations:** 1 Department of Pharmaceutical Services, 8785 UCSF Health, San Francisco, CA, USA; 2 1792 St. Luke's Cancer Institute, Boise, ID, USA; 3 Department of Pharmacy, 205134 Montefiore Medical Center: Einstein Campus, Bronx, NY, USA

**Keywords:** Tagraxofusp, CD123, BPDCN, administration

## Introduction

Tagraxofusp (TAG), a first-in-class CD123-targeted therapy, is the only drug approved in the United States (U.S.) for adults or pediatric patients aged ≥2 years with treatment-naïve or relapsed/refractory blastic plasmacytoid dendritic cell neoplasm (BPDCN).^1^ In first-line BPDCN treatment, TAG is associated with high rates of rapid and durable responses (median of 39 days and median duration of complete response [CR]/CR with residual skin abnormalities not indicative of the active disease [CR/CRc] of 24.9 months, respectively), including 51% of CR/CRc patients bridging to stem cell transplantation.^2^ TAG has a safety profile that is well-characterized and manageable, with adverse events most often occurring in cycle 1 and are transient, with no cumulative long-term toxicity and no myelosuppression.^2^ TAG is administered intravenously at a dose of 12 µg/kg/day on days 1–5 of a 21-day cycle. It is required that the first cycle of TAG be administered in an inpatient, hospital setting, and that the patient remain in the hospital to be monitored for at least 24 h following the last dose of cycle 1. After the successful administration of cycle 1, patients can transition to appropriate outpatient facilities to receive cycle 2 and beyond.

**Figure 1.**
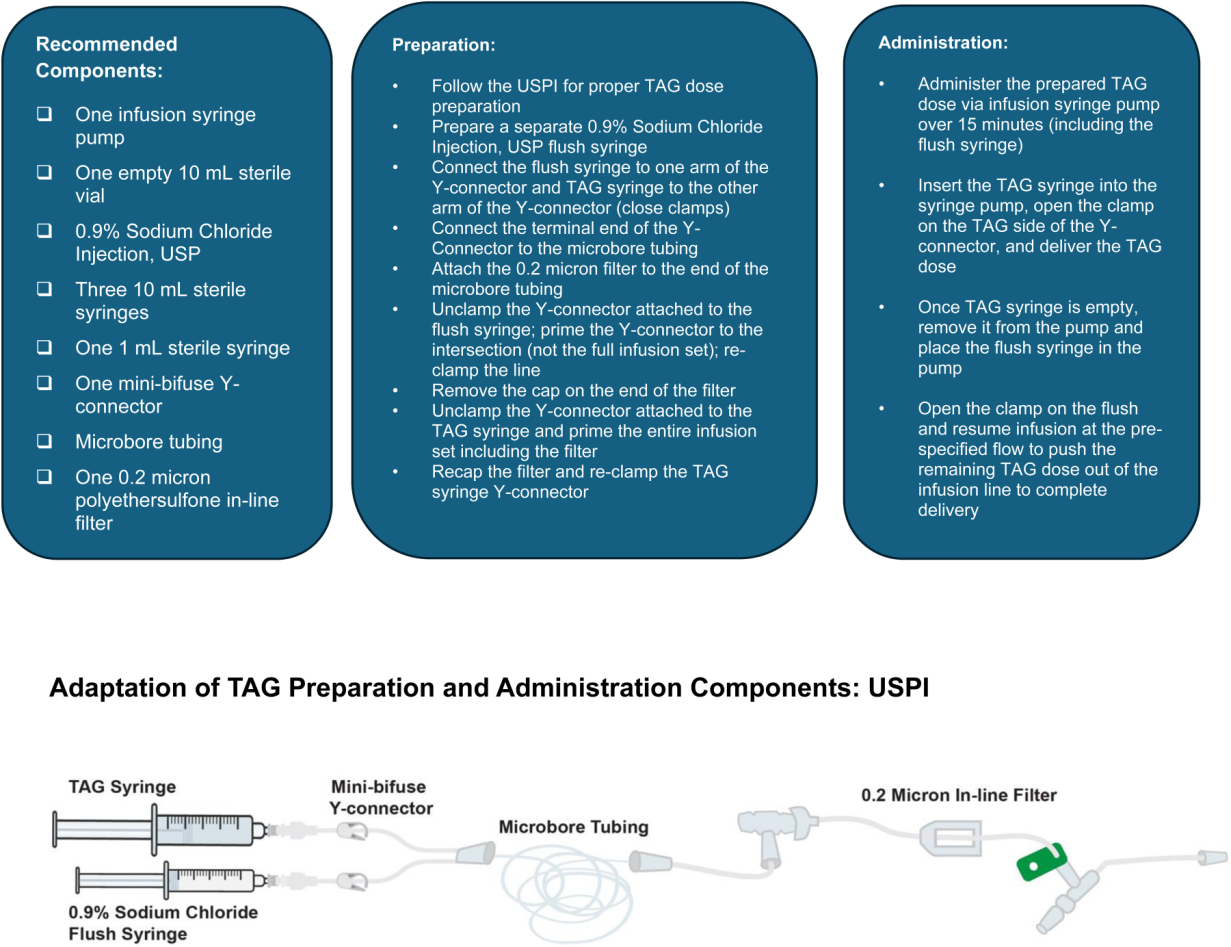
USPI preparation and administration of TAG.

The U.S. prescribing information (USPI) for TAG outlines specific components required for dose preparation and administration (Figure 1).^1^ As BPDCN is an aggressive disease, it is essential to prevent delays in treatment administration that can occur if these TAG-specific administration components are not readily available. Herein, we describe alternative administration techniques utilized by three healthcare facilities to help expedite the appropriate selection and safe administration of TAG.

## Alternative components and techniques

### Technique #1

**Figure 2.**
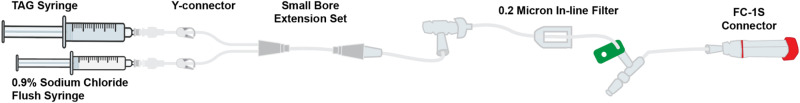
Adaptation of TAG preparation and administration components: technique #1.

Of the three alternative component administration techniques described, technique #1 (Figure 2) most closely follows the USPI. Dose preparation proceeds without the use of closed-system transfer devices (CSTDs) in accordance with the TAG USPI. The TAG dose (in a 10 mL–20 mL syringe based on dose) and 0.9% sodium chloride flush are prepared by pharmacy and connected to the mini-bifuse Y-connector (small-bore bifuse extension set with two Remv MicroClave^TM^ clear, two clamps, rotating leur; Reference MC33081), microbore tubing (ICU Medical, Smallbore Extension Set w/Clamp NanoClave^TM^ T-Connector, rotating luer; Ref A1001), and a 0.2 micron polyethersulfone in-line filter (ICU Medical, 14” Ext set with 0.2 micron filter; Ref MC9013). The Y-connector and infusion set are primed per the USPI. An Equashield^®^ female luer lock connector (Ref FC-1S) is added to the end of the filter extension set for connection to the patient to help decrease the risk of exposure during administration. Prior to connecting the medication to the patient for administration, the TAG dose is confirmed by nursing. The TAG dose is administered over 15 min followed by the 0.9% sodium chloride flush. Technique #1 differs from the TAG USPI in that a CSTD is added at the end of the filter extension set and instead of administering both the TAG dose and flush over 15 min, the TAG dose is given over 15 min, and the flush is delivered subsequently at the same rate.

### Technique #2

**Figure 3.**
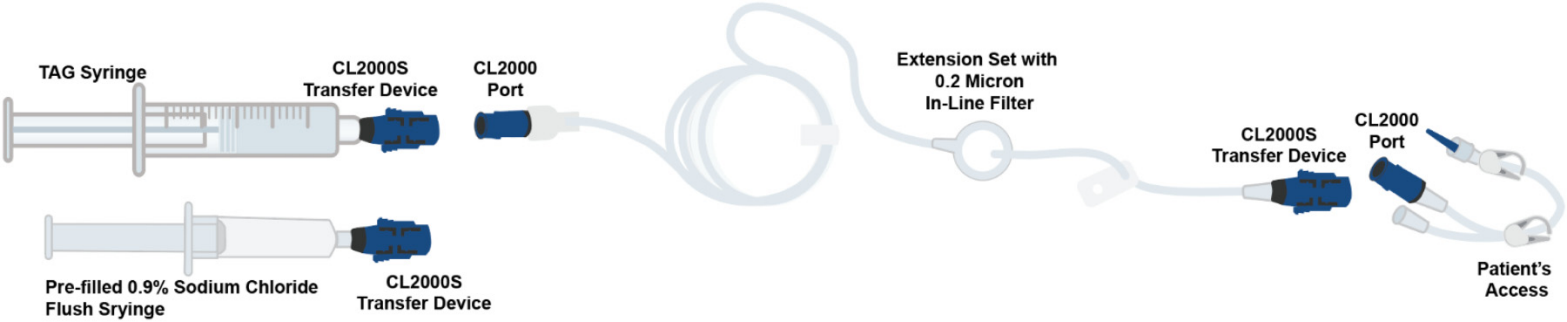
Adaptation of TAG preparation and administration components: technique #2.

The second alternative components technique (Figure 3) begins with preparation of TAG following the USPI but introduces the use of ICU Medical ChemoLock^TM^ CSTD throughout the compounding and administration process (Refs CL-80S vial spike, CL2100 port, CL2000S transfer device). Once pharmacy has prepared the BD 30 mL TAG syringe with a CL2000S transfer device, the syringe is dispensed for nurse verification without connection to the microbore tubing or 0.2-micron filter. The nurse verifies the dose in the syringe against the order per the institute's standard of practice (SOP), then connects the microbore tubing with a built-in in-line filter (BD extension set microbore tubing with 0.2-micron low protein binding filter; Ref 10010570) that has been pre-primed with 0.9% sodium chloride injection. The nurse attaches a CL2000S transfer device to the male luer on the extension set and a CL2000 port to the female luer of the extension set. The TAG syringe is connected to the extension set via the CL2000 port. After a two-nurse dose verification is completed chairside, the CL2000S on the extension set is connected to the CL2000 port on the patient's access. The Alaris syringe pump is programmed to deliver the TAG syringe, and once emptied, it is removed from the pump by disconnecting the CSTD, and a pre-filled 10 mL 0.9% sodium chloride injection flush syringe is attached to a CL2000S transfer device and placed in the pump. Variances from the TAG USPI include the following: a Y-connector is not used; the tubing is primed with 0.9% sodium chloride versus drug solution; ICU Medical ChemoLock CSTDs are used throughout; and the nurses use a prefilled 10 mL 0.9% sodium chloride flush instead of a flush dispensed from pharmacy.

### Technique #3

**Figure 4.**
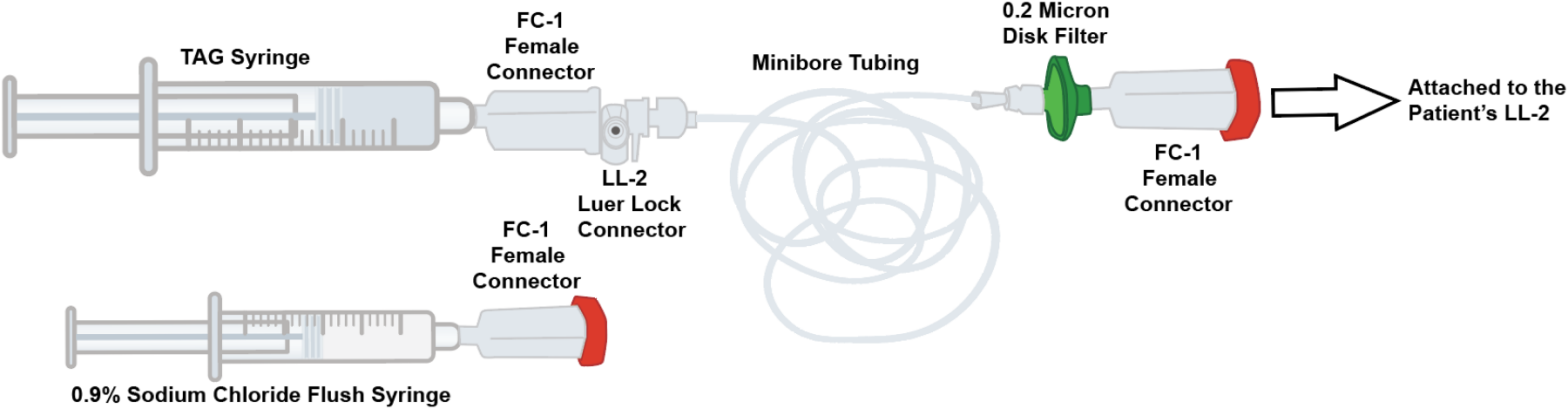
Adaptation of TAG preparation and administration components: technique #3.

Technique #3 uses a TAG preparation and administration process (Figure 4) that differs slightly from the USPI. The preparation and administration of TAG involve the use of Equashield^®^ CSTD. Although Equashield syringes are also typically used with the CSTD connectors at this institution, BD syringes are explicitly used for TAG preparation and administration due to syringe pump compatibility. It is important for each institution to ensure that all TAG-specific administration components are compatible with the designated syringe pump. The pharmacy prepares the TAG dose in a 30 mL sterile BD syringe with an Equashield female connector (Ref FC-1) and attaches the microbore tubing (Medline 60” IV Extension Set; Ref DYNDTN0007) via an Equashield Luer Lock connector (Ref LL-2). A 0.2-micron disk filter (BBraun 0.2 micron disk filter; Ref 415002) is attached to the opposite end of the microbore tubing, and an Equashield FC-1 is added to the end of the disk filter; the tubing is primed with the drug solution. A 3 mL 0.9% sodium chloride flush is prepared, and an Equashield FC-1 is added to the syringe. The TAG administration set and the saline flush are dispensed for nurse verification. The medication is attached to the patients’ access via an Equashield LL-2 and delivered via a syringe pump. Key variations in this technique versus the USPI include not utilizing a Y-connector and use of Equashield CSTDs during preparation and administration.

## Discussion

Providers should aim to prioritize using the preparation and administration components outlined in the TAG USPI (eg, a mini-bifuse y-connector, a 0.2-micron polyethersulfone in-line filter, microbore tubing, etc). When availability of any of the USPI components poses a challenge to timely treatment, the techniques described herein offer workable alternative administration solutions. TAG is currently not categorized as a hazardous drug by the National Institute for Occupational Safety and Health (NIOSH),^3^ allowing institutions flexibility to follow their internal policy for preparation and administration. However, risk awareness is advised. The details from the processes explained within this article can help to alleviate some of the challenges that US healthcare providers may face in expediting treatment administration for patients with BPDCN. These TAG administration techniques have not yielded unexpected adverse effects or safety concerns to date. Since TAG is the only approved therapy for BPDCN, having alternative components and processes for preparation and administration will ensure that patients receive the desired treatment in a timely manner.

## References

[1] Stemline Therapeutics Inc. Elzonris® (package insert). New York: Stemline Therapeutics Inc, 2023.

[2] Pemmaraju N Sweet KL Stein AS , et al. Long-Term benefits of tagraxofusp for patients with blastic plasmacytoid dendritic cell neoplasm. J Clin Oncol 2022; 40: 3032–3036. 35820082 10.1200/JCO.22.00034 PMC9462530

[3] NIOSH [2024]. NIOSH list of hazardous drugs in healthcare settings, 2024. 10.26616/NIOSHPUB2025103 (2024, accessed 31 March 2025).

